# Assessment of Tumor Mutational Burden in Pediatric Tumors by Real-Life Whole-Exome Sequencing and In Silico Simulation of Targeted Gene Panels: How the Choice of Method Could Affect the Clinical Decision?

**DOI:** 10.3390/cancers12010230

**Published:** 2020-01-17

**Authors:** Hana Noskova, Michal Kyr, Karol Pal, Tomas Merta, Peter Mudry, Kristyna Polaskova, Tina Catela Ivkovic, Sona Adamcova, Tekla Hornakova, Marta Jezova, Leos Kren, Jaroslav Sterba, Ondrej Slaby

**Affiliations:** 1 Central European Institute of Technology, Masaryk University, 62500 Brno, Czech Republic; hana.noskova@ceitec.muni.cz (H.N.); pal@mail.muni.cz (K.P.); tina.ivkovic@ceitec.muni.cz (T.C.I.); sona.adamcova@ceitec.muni.cz (S.A.); tekla.hornakova@hotmail.com (T.H.); 2 Department of Pediatric Oncology, University Hospital Brno, 613 00 Brno, Czech Republic; kyr.michal2@fnbrno.cz (M.K.); merta.tomas@fnbrno.cz (T.M.); Mudry.Peter@fnbrno.cz (P.M.); polaskova.kristyna@fnbrno.cz (K.P.); 3 Faculty of Medicine, Masaryk University, 62500 Brno, Czech Republic; 4 International Clinical Research Center, St. Anne’s University Hospital, 65691 Brno, Czech Republic; 5 Department of Hematology, University Hospital Schleswig-Holstein, 24105 Kiel, Germany; 6 Department of Pathology, University Hospital Brno, 62500 Brno, Czech Republic; jezova.marta@fnbrno.cz (M.J.); Kren.Leos@fnbrno.cz (L.K.); 7 Regional Centre for Applied Molecular Oncology, Masaryk Memorial Cancer Institute, 60200 Brno, Czech Republic

**Keywords:** pediatric tumors, tumor mutational burden, TMB, whole-exome sequencing, gene panel sequencing, immune checkpoint inhibitors

## Abstract

Background: Tumor mutational burden (TMB) is an emerging genomic biomarker in cancer that has been associated with improved response to immune checkpoint inhibitors (ICIs) in adult cancers. It was described that variability in TMB assessment is introduced by different laboratory techniques and various settings of bioinformatic pipelines. In pediatric oncology, no study has been published describing this variability so far. Methods: In our study, we performed whole exome sequencing (WES, both germline and somatic) and calculated TMB in 106 patients with high-risk/recurrent pediatric solid tumors of 28 distinct cancer types. Subsequently, we used WES data for TMB calculation using an in silico approach simulating two The Food and Drug Administration (FDA)-approved/authorized comprehensive genomic panels for cancer. Results: We describe a strong correlation between WES-based and panel-based TMBs; however, we show that this high correlation is significantly affected by inclusion of only a few hypermutated cases. In the series of nine cases, we determined TMB in two sequentially collected tumor tissue specimens and observed an increase in TMB along with tumor progression. Furthermore, we evaluated the extent to which potential ICI indication could be affected by variability in techniques and bioinformatic pipelines used for TMB assessment. We confirmed that this technological variability could significantly affect ICI indication in pediatric cancer patients; however, this significance decreases with the increasing cut-off values. Conclusions: For the first time in pediatric oncology, we assessed the reliability of TMB estimation across multiple pediatric cancer types using real-life WES and in silico analysis of two major targeted gene panels and confirmed a significant technological variability to be introduced by different laboratory techniques and various settings of bioinformatic pipelines.

## 1. Introduction

The cancer cell genome acquires genetic alterations differing from the germline of the host [1]. Somatic mutation rates can be affected by exposure to exogenous factors, such as ultraviolet light or tobacco smoke [2], or by compounding genetic defects, such as DNA mismatch repair deficiency, microsatellite instability, or replicative DNA polymerase mutations [1,2,3]. These somatic genetic alterations induce and drive carcinogenesis. The type and the number of acquired mutations varies among the cancer types but also among the affected individuals [4]. Some of these mutations lead to the formation of tumor-specific neoantigens, which could be recognized by a patient’s immune system as non-self and which are highly clinically relevant since these neoantigens can make the cancer cells sensitive to treatment with immune checkpoint inhibitors (ICIs) against cytotoxic T-lymphocyte-associated protein 4 (CTLA-4), programmed cell death protein 1 (PD-1) and programmed death-ligand 1 (PD-L1) in various cancers including melanoma [5], non–small-cell lung cancer (NSCLC) [6], kidney cancer [7], bladder cancer [8] and others [9]. The genomic landscape of smoking-induced NSCLC and UV light-induced melanoma is often characterized by a high number of acquired alterations, while leukemias and pediatric tumors show the lowest mutations counts.

Rapidly developing genomic methods based on next-generation sequencing (NGS) simplified the detection and quantification of these acquired changes on the level of individual cancer genomes. Tumor mutational burden (TMB) is a quantitative measure of acquired somatic mutations in the cancer cell genome. Initial exploratory analyses of TMB in cancer patients [10,11] were carried out using whole exome sequencing (WES). WES is a comprehensive research tool for assessment of genomic alterations across the entire coding region of the ~22,000 genes in the human genome, comprising of 1–2% of the genome [3,12]. Currently, WES-derived TMB values are considered to be the gold standard, but the high cost and long turnaround time limit routine diagnostic applicability of WES. Therefore, targeted NGS cancer gene panels have been promoted for TMB estimation as a feasible and cheaper alternative to WES [13]. Whereas TMB assessed by WES is typically reported as the total number of mutations per cancer cell exome, TMB assessed by gene panel assays is usually referred to as mutations per megabase (mut/Mb) because it differs in the number of genes and target region size [2,3,14]. The precise calculation of TMB may, however, vary depending on the region of tumor genome sequenced, types of mutations included, methods of subtracting germline variants and other aspects of bioinformatic analysis pipeline of the sequencing data [3,15]. Both the FDA-approved FoundationOne CDx (F1CDx) panel and the FDA-authorized Memorial Sloan Kettering-Integrated Mutation Profiling of Actionable Cancer Targets (MSK-IMPACT) panel used correlation between panel- and WES-based TMB to validate the reliability of panel based TMB estimation, and they claimed that these panels can assess TMB accurately (R = 0.74 for F1CDx and R = 0.76 for MSK-IMPACT) [2,13,16]. However, as Wu et al. [13] proposed in their recent work, the overall correlation between the panel- and WES-based TMB could be substantially distorted by outliers (i.e., cases with relatively ultra-high TMB within each cancer type) [13], which might lead to overestimation of the reliability of panel-based TMB estimation. Therefore, additional studies are needed to evaluate the significance of correlation between the WES-based and targeted panel-based TMB values.

As already mentioned, TMB is considered to be a proxy for cancer cell neo-antigenicity and therefore could potentially serve as a predictive biomarker of therapeutic response to ICI. Several studies, especially in NSCLC, retrospectively employed WES or larger NGS panels to determine TMB as a potential response predictor [17,18,19]. Unfortunately, the definition of cut-off values to separate “high TMB” from “low TMB” tumors is not consistent in recent NSCLC trials. For example, in the CheckMate (CM) trials CM012 (nivolumab and ipilimumab) [20], CM227 (nivolumab and ipilimumab) [17] and CM026 (nivolumab only) [21] cut-points of 158 mutations, 199 mutations and 243 somatic missense mutations (number of mutations estimated from a commercial gene panel based cut point of 10 mutations per Mbp) were used, respectively [22].

This is the first study in pediatric oncology that aims to assess the reliability of TMB estimation using real-life WES across multiple cancer types and in silico analysis of two major gene panels, which are widely used for routine diagnostics in clinical practice, where various settings of bioinformatic pipeline were employed. The performance and correlation of WES and panel-based TMB assessment methods were evaluated together with potential consequences for clinical decision making where various cut-offs for ICI indication were used.

## 2. Results

### 2.1. Comparison of TMB between Real-Life WES and In Silico Targeted Gene Panels

**Figure 1:**
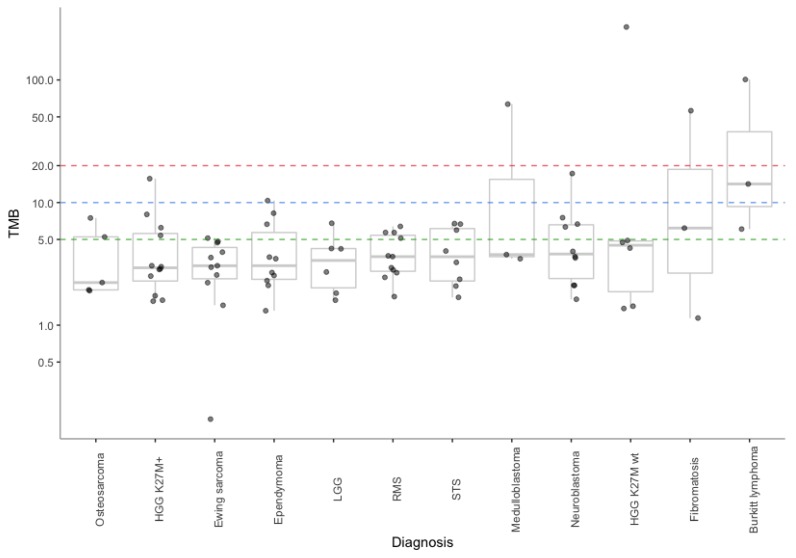
Tumor mutational burden (TMB) values determined in our pediatric cancer patient cohort (WES—Method1) stratified by cancer type. Hypothetical TMB cut-off values are shown as dashed lines (green, TMB ≥ 5; blue, TMB ≥ 10, red, TMB ≥ 20).

We successfully performed germline and somatic WES and calculated TMB in 106 pediatric patients of 28 distinct cancer types. We stratified patients based on their diagnosis and expressed TMB for each group of patients as a median (min–max) or as a concrete value in cases where there was only one patient within a group (summarized in Table 1). WES-based TMB for each tumor is depicted in Figure 1. The median TMB ranged widely among diagnoses, from 0.3 mutations/Mb in myeloid sarcoma to 14.2 mutations/Mb in Burkitt lymphoma. 

**Figure 2:**
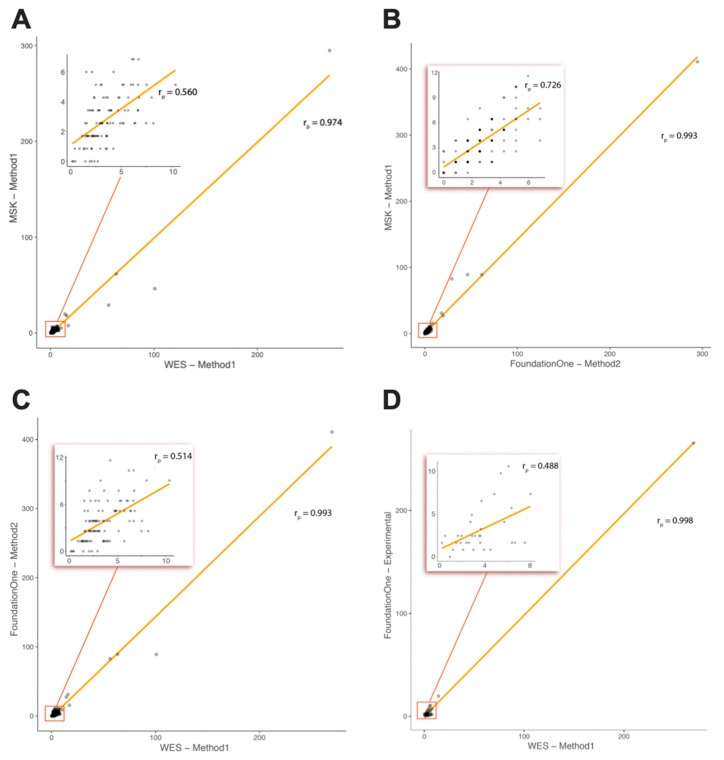
Correlation of tumor mutational burden (TMB) determined by real-life WES and targeted gene panels: real-life WES vs. in silico MSK-IMPACT (**A**), in silico F1CDx vs. MSK-IMPACT (**B**), real-life WES vs. in silico F1CDx (**C**), real-life WES vs. real-life laboratory service F1Heme (**D**).

Furthermore, we determined, by an in silico approach, whether TMB, as measured by WES, correlates with TMB calculated by the gene sets and bioinformatic approaches used by two commercially available targeted gene panels. Panel-based TMB (MSK-IMPACT and F1CDx) for each group of patients expressed as a median (min–max) or as a concrete value in cases where there was only one patient in a group are summarized in Table 2. We confirmed a strong Pearson correlation of the panel TMB with the WES-based TMB characterized by R = 0.993 (F1CDx), and R = 0.974 (MSK-IMPACT), respectively (Figure 2A,C). Correlation between MSK-IMPACT and F1CDx panels was R = 0.993 (Figure 2B). The TMB assessment method was adapted for each panel accordingly (MSK-IMPACT—Method 1; F1CDx—Method 2). However, when the few hypermutated cases were excluded and only samples with TMB <10 mut/Mb were considered for analysis, the correlation decreased significantly: R = 0.514 (F1CDx), and R = 0.560 (MSK-IMPACT). Correlation between TMBs determined by the two panels remained remarkably higher (R = 0.726).

### 2.2. Comparison of TMB between Real-Life WES and the Foundation Medicine Inc. (FMI) Testing Service (Subcohort of Patients)

In the subgroup of 34 patients (randomly selected from the patients where a Formalin-Fixed Paraffin-Embedded (FFPE) block with tumor tissue was available), comparative study of real-life WES-based TMB assessment and the FMI testing service was performed. For the WES samples, tumor and normal tissue were each sequenced in order to distinguish germline polymorphisms from somatic mutations. For the targeted FMI testing, no matched normal material was sequenced; rather, genomic variants were stringently filtered to eliminate germline polymorphisms, as declared by the vendor. For TMB determination from WES data, we used Method 1 (excluding indels and synonymous mutations). The FMI testing services are done using Method 2 (including indels and synonymous mutations). In nine cases, different samples from one resection or biopsy collection were used. This is summarized in Table 2. However, the Pearson correlation between TMBs determined by these two real-life approaches was comparable to the correlation of real-life WES and in silico F1CDx panel (R = 0.998 vs. R = 0.993) indicating the relevance of the in silico approach for TMB assessment comparative studies. When hypermutated cases were excluded, correlation decreased to R = 0.488 (Figure 2D), which is similar to the decrease observed in the in silico approach (R = 0.514).

### 2.3. WES-Based TMB Values during Tumor Progression

In nine cases, we determined the TMB by WES in sequential tumor biopsies or tumor tissues from surgical resection. In five cases, we used tumor tissue from a primary tumor and its relapse. In the remaining four cases, tumor tissue was collected from two consequent local or metastatic relapses. TMB values are summarized in Table 3. In seven out of nine cases, an increase in TMB in the second tumor tissue was observed, with the average increase being 1.6 ± 1.3 mut/Mb.

### 2.4. Consequence of TMB Assessment Method for ICI Indication

TMB as a predictive biomarker is currently the focus of several clinical trials with ICI. We have evaluated how the sequencing region (WES vs. the gene set used in MSK-IMPACT vs. the gene set used in F1CDx) and method for TMB calculation affect the final TMB and potential ICI indication when various hypothetical cut-off values are applied. Results of this analysis are summarized in Table 4. As expected, the number of patients above a cut-off is always higher with WES-based TMB assessment (compared to panel-based) and when TMB is assessed by Method 2 (including indels and synonymous mutations). Number of patients above a cut-off differs significantly when low TMB cut-off value is applied (cut-off ≥ 5). With the increasing cut-off values, the significance of technological variability introduced by sequencing various genome regions and different TMB calculating methods decreases. However, even with a relatively high cut-off value (cut-off ≥ 20), the number of pediatric patients hypothetically indicated for ICI therapy differs between TMB groups calculated with Method 1 and Method 2 (e.g., four vs. seven pediatric patients with WES).

## 3. Discussion

The predictive power of TMB as a biomarker for response to ICI is currently being investigated in many clinical trials across various cancer types. Patients with a higher TMB are more likely to respond to ICI in various settings, including PD-(L)1 blockade in NSCLC [10], CTLA-4 blockade in malignant melanoma [11], and combined PD(L)-1 and CTLA-4 blockade in NSCLC [17]. Studies have shown that TMB is to a large extent independent of the PD-L1 status and might thereby identify additional subgroups of patients who benefit from ICI [17,20,22].

Based on these clinical observations, TMB became an emerging predictive biomarker for ICI in various cancer types, and an urgent need occurred to answer the questions concerning the technological aspects affecting TMB detection by WES and targeted panel sequencing to ensure implementation of lab developed tests that guarantee optimal reference standard quality for patient stratification [19].

In initial studies, WES was widely used to determine TMB and is still considered to be the gold standard; however, targeted sequencing panels are more readily interpretable and are a more pragmatic and potentially cost-effective approach to TMB testing in clinical diagnostics [3]. While in the context of clinical trial, TMB testing is mainly carried out by commercial vendors, many clinical laboratories depending on the regulatory approval context may eventually use in-house designed panels to determine TMB scores [22]. Endris and others have already investigated the minimum required size of a gene panel by comprehensive in silico analyses of available WES data sets and have shown that at least 1 Mbp of exonic and/or intronic region should be sequenced to achieve a similar power in discriminating ICI responders from non-responders comparable to WES [19]. Furthermore, Buchhalter at al. showed that “size does matter”, with an optimal panel size being between 1.5 and 3 Mbp, considering the benefit–cost ratio, and that the inclusion of all point mutations (instead of only missense mutations) in the TMB calculation is possible and recommendable to enhance precision [9].

In our study, we focused on the potential technological variability introduced to TMB scoring by the usage of various platforms and bioinformatic pipelines for their assessment in pediatric tumors. As a reference method, we performed WES and subsequently in silico simulated two most frequently used sequencing panels, MSK-IMPACT and F1CDx. We confirmed a strong Pearson correlation of the panel-based TMB with the WES-based TMB; however, when the few hypermutated cases were excluded and only samples with TMB < 10 mut/Mb were considered for analysis, the correlation decreased significantly (Figure 2). This indicates a significant bias introduced to correlation analysis by only a few hypermutated cases included in the study. Correlation between samples with TMB < 10 mut/Mb was not satisfactory and probably lead to significant clinical misclassifications in the routine diagnostic scenario based on the usage of a cut-off value in the range of 5 to 15 mut/Mb. Similar observations were also provided by other authors describing adult tumors [9,19].

In a subgroup of patients, we performed a comparative study of real-life WES-based TMB assessment and the FMI testing service where we observed a similar effect of the hypermutated cases on the correlation significance. In agreement with others [9,19], we observed that the identification of high TMB tumors can be reliably achieved by any of the tested methods (cases with ultra-hypermutated tumors). However, the vast majority of tumors have intermediate TMB values; in these cases, a technological variability interferes with the reliable differentiation between TMB-high and low tumors [9,19].

In nine cases, we determined the TMB by WES in sequential tumor biopsies or tumor tissues from surgical resection. As expected, in seven out of nine cases, there was an increase in TMB in the second tumor with the average increase being approx. 2 mut/Mb. Surprisingly, in two cases, we observed a decrease in TMB, which could be explained mainly by the quality of the tumor tissue specimen and a low content of tumor cells in the second tumor which could decrease detectable mutations used for TMB assessment. It is important to mention that tumor content in the tissue specimens is an important factor affecting TMB scoring and is often not considered in TMB studies. 

Finally, we evaluated how the sequencing region (WES vs. the gene set used in MSK-IMPACT vs. the gene set used in F1CDx) and the bioinformatic pipeline used for TMB calculation affect the final TMB and potential ICI indication when various hypothetical cut-off values are applied. In general, as expected, the number of patients above a cut-off is always higher in WES-based TMB assessment (compared to panel-based) and when the TMB is assessed by Method 2 (including indels and synonymous mutations). We also found that with the increasing cut-off values, the significance of technological variability and consequent clinical misclassification decreases. However, certain combinations of settings of TMB assessment methods (e.g., WES-M2 vs. F1CDx-M1), compounded by the use of a cut-off value of 10 mut/Mb, yield extremely different results. While the first approach predicts 25 patients to be good responders to ICI, the second approach predicts only seven patients. This indicates a potentially very strong misclassification issue for routine diagnostics. Based on the currently available results from clinical trials, it is very difficult to judge whether TMB assessed by Method 1 or Method 2 is a more accurate predictive biomarker of response to ICI therapy. Unfortunately, this in silico modeling has not been performed in the context of clinical outcomes from ICI trials.

## 4. Materials and Methods 

### 4.1. Patients and Biological Specimens

We reviewed tumor mutational burden (TMB) results from 106 patients with pediatric high-risk/recurrent solid tumors (both newly diagnosed and relapsed) who had undergone laboratory WES at Central European Institute of Technology (CEITEC, Masaryk University, Brno, Czech Republic). Informed consent was obtained from all patients and all experiments using clinical samples were performed in accordance with the approved international guidelines. After surgical resection of the tumor or collection of the tumor biopsies, tissue samples were evaluated by an experienced surgical pathologist for the tumor cell content, and only specimens with more than 20% of the tumor cells were included. In addition, peripheral blood was collected to obtain DNA for germline WES. Number of patients stratified according to their diagnoses and related clinical data are summarized in Table 5. In nine cases, we collected two consequent tissue specimens (diagnosis/relapse or two relapses) and both were used for WES and TMB assessment.

### 4.2. DNA Isolation

Tumor DNA was extracted from the FFPE samples or fresh frozen tissues using QIAmp DNA FFPE Tissue Kit (Qiagen, Venlo, The Netherland) or QIAamp DNA Micro Kit (Qiagen). Germline DNA was extracted from peripheral blood leukocytes using QIAamp DNA Micro Kit (Qiagen). The purified DNA was quantified using Qubit 2.0 Fluorometer and NanoDrop 2000c spectrophotometer (both Thermo Fisher Scientific, MA, USA).

### 4.3. Whole Exome Sequencing

Libraries for whole exome capture and sequencing were prepared using TruSeq Exome Kit (Illumina, CA, USA) according to manufacturer´s recommendations. Quantity and quality of the exome libraries were checked using Qubit 2.0 Fluorometer and NanoDrop 2000c spectrophotometer (Thermo Fisher Scientific). Prepared libraries were loaded onto NextSeq 500/550 Mid Output Kit (150 cycles) and sequenced on the NextSeq 500 instrument (both Illumina). Sequencing coverage for both exomes was >20 × at >90% of captured regions.

### 4.4. Bioinformatic Analysis

**Figure 3:**
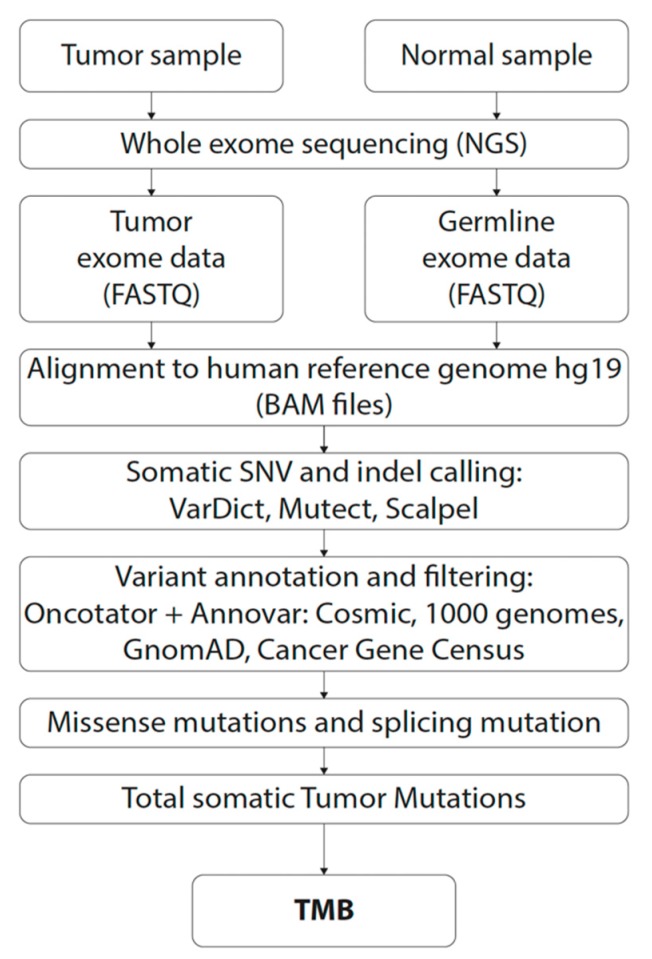
Workflow for tumor mutational burden (TMB) assessment by WES in this study.

Sequencing reads in FASTQ format were mapped to the human reference genome hg19 with the BWA-MEM algorithm [23] for both the tumor and the healthy control sample. The resulting alignments in BAM format were postprocessed with the SAMBLASTER program [24] for marking PCR duplicates. The final alignment file of the control sample was used to assess single nucleotide variants (SNVs) and short insertions/deletions (indels). Two variant callers were used for germline variant calling; the GATK HaplotypeCaller [25] and VarDict [26]. Reported variants were annotated with Annovar [27] and Oncotator [28] annotation programs. Tumor specific variants were assessed by somatic (paired; tumor vs. control) variant calling. For this purpose, we used GATK MuTect2 (SNVs), Scalpel [29] (Indels), and VarDict (SNVs and Indels) variant callers. The annotation of somatic variants was performed with the addition of the COSMIC database [30]. Overview of the bioinformatic pipeline is depicted in Figure 3.

### 4.5. Tumor Mutational Burden Estimation

An annotated list of somatic variants from the previous step was used to assess the TMB. We chose to compare two methods of TMB estimation, both based on publicly available approaches.

Method 1 (M1)—In our laboratory, we only consider somatic single nucleotide variants (SNVs) for TMB calculation from WES data, since indels (short insertions and deletions) tend to be called with high false positive rates and could potentially skew the outcome. Additionally, two bases before and after each exon are considered as splicing mutations. Synonymous variants are filtered out, as they do not fit the definition of TMB. Finally, variants with variant allele frequency (VAF) of less than 5% are also filtered out. This approach is also used by MSK-IMPACT NGS panel.

Method 2 (M2)—This approach, used by the Foundation Medicine Inc. (FMI) targeted panels (e.g., F1CDx [2] as well as F1Heme), defines TMB as the number of SNVs (including synonymous variants) and indels in the coding regions of targeted genes. However, splicing variants are not included. A 5% cut-off for the VAF was also applied.

For the final TMB calculation, in both methods, the sum of variants remaining after application of the all filters, is then divided by the size (in megabases) of the target region from which the variants have been assessed. The target regions together with their sizes are listed below.

Both methods were applied to the three target regions (as shown in Table 5):

1. All coding sequences (whole exome; 35 Mb; using M1 for TMB calculation);
2. The coding sequences of genes analyzed by the FMI (F1CDx panel; 324 cancer-related genes; 0,8 Mbl using M2 for TMB calculation);
3. The coding sequences of genes analyzed by the Memorial Sloan Kettering Cancer Center (MSK-IMPACT; 468 cancer-related genes; 1.22 Mb; using M1 for TMB calculation)

The coding region locations on the hg19 genome were downloaded from the UCSC web site.

### 4.6. Comparative Study with the Foundation Medicine Inc. (FMI) Sequencing Service

FFPE tumor tissue samples of 34 patients who were previously examined by WES in our laboratory and were sent to the FMI for the FoundationOne Heme (F1Heme) test, which is recommended by vendor for pediatric tumors. In the nine cases, WES was performed using fresh frozen tissue, while different FFPE samples were sent for the F1Heme test. These specimens are indicated in the summarizing tables (Table 3) with the TMB results.

## 5. Conclusions

We present a study, where, for the first time in the context of pediatric tumors, the reliability of TMB estimation across multiple pediatric cancer types using real-life WES and in silico analysis of two major targeted gene panels was assessed. We confirmed a significant technological variability introduced by different laboratory technologies and various settings of bioinformatic pipelines. These results may provide valuable information for improving the accuracy of TMB estimation based on targeted gene panel sequencing in a diagnostic setting. Our study confirmed previous observations from adult tumors and thus supports the incentive to establish concordance between assay platforms used across different clinical trials in order to achieve a successful real-world implementation of TMB testing. To this end, worldwide efforts to ensure the harmonization of TMB assessment are ongoing [31,32,33].

## Figures and Tables

**Table 1:** Comparison of TMB determined by real-life WES and in silico targeted gene panels.

Diagnosis	TMB WES—M1 * Real-Life (Median/Value)	(Min–Max)	TMB MSK—M1 * In Silico (Median/Value)	(Min–Max)	TMB F1CDx—M2 ** In Silico (Median/Value)	(Min–Max)
HGG glioma H3K27M+	2.9	(1.6–15.7)	4.7	(2.6–17.9)	4.5	(2.6–31)
Rhabdomyosarcoma	3.6	(1.7–6.4)	2.6	(1.7–4.3)	2.6	(0–5.2)
Ewing sarcoma	3.1	(0.2–5.1)	2.6	(0–5.1)	2.6	(0–7.8)
Ependymoma	3.1	(1.3–10.4)	1.7	(0–5.1)	3.2	(1.3–9)
Neuroblastoma	3.8	(1.6–17.2)	3.0	(0.9–7.7)	4.5	(1.3–15.5)
Soft tissue sarcoma	3.6	(1.7–6.7)	3.4	(0–6.8)	3.2	(0–9)
Low-grade glioma	3.5	(1.6–6.8)	2.1	(0.9–4.3)	3.9	(1.3–5.2)
High-grade glioma H3K27M wt	4.5	(1.4–269.8)	3.4	(0.9–294.7)	5.2	(1.3–410.9)
Osteosarcoma	2.2	(1.9–7.5)	3.4	(0–5.1)	5.2	(1.3–6.5)
Burkitt lymphoma	14.2	(6.1–100.7)	19.6	(6.8–46.1)	27.1	(6.5–89.2)
Medulloblastoma	3.8	(3.5–63.6)	3.4	(0.9–61.5)	3.9	(1.3–89.2)
Fibromatosis	6.2	(1.1–56.2)	5.1	(1.7–29)	10.3	(1.3–82.7)
Wilms tumor	3.1	(2.3–3.9)	3.4	(2.6–4.3)	2.6	(1.3–3.9)
Renal cell carcinoma	1.8	(1.5–2.1)	4.3	(2.6–6.0)	4.5	(1.3–7.8)
Adrenocortical carcinoma	0.9	-	0.9	-	1.3	-
Plexus choroideus carcinoma	5.2	-	2.6	-	5.2	-
Hepatocellular carcinoma	3.6	-	0.9	-	3.9	-
Disseminated adenocarcinoma	2.3	-	4.3	-	6.5	-
Familiar infantile myofibromatosis	2.1	-	1.7	-	0.0	-
Myeloid sarcoma	0.3	-	0.0	-	0.0	-
Undifferentiated embryonal tumor of spinal canal	3.1	-	2.6	-	2.6	-
Nongerminomatous Germ Cell tumor CNS	2.3	-	1.7	-	1.3	-
Epithelial hepatoblastoma	0.5	-	0.0	-	0.0	-
Spindle cell hemangioma	2.1	-	0.9	-	2.6	-
Fibrodysplasia ossificans progressiva	3.1	-	2.6	-	2.6	-
Hepatosplenic T-lymphoma	0.4	-	0.9	-	0.0	-
Multisystemic Langerhans cell histiocytosis	3.1	-	2.6	-	3.9	-
Gastrointestinal stromal tumor	2.7	-	3.4	-	6.5	-

* M1—Method 1 for calculation of TMB excluding synonymous variants and indels; ** M2—Method 2 for calculation of TMB including synonymous variants and indels.

**Table 2:** Comparison of TMB determined by real-life WES and the FMI laboratory testing service FoundationOne Heme (F1Heme).

Gender	Age at Diagnosis	Diagnosis	TMB F1Heme Real-Life (Mut/Mb)	TMB WES—M1 * Real-Life (Mut/Mb)	Same Sample (Yes/No)
F	9	Renal cell carcinoma	1.63	1.45	yes
F	7	Diffuse intrinsic pontine glioma H3K27M+	2.44	1.60	yes
M	13	Desmoid fibromatosis	0.81	1.14	yes
M	6	Spindle cell hemangioma	0.81	2.05	yes
F	14	Gastrointestinal stromal tumor	4.07	2.71	yes
F	14	Osteosarcoma	2.44	1.91	yes
M	2	Langerhans cell histiocytosis	2.44	3.11	yes
M	11	Wilms tumor	1.63	2.34	yes
M	11	Ewing sarcoma	1.63	2.57	yes
F	7	Ependymoma	2.44	3.48	yes
M	18	Embryonal rhabdomyosarcoma	4.89	2.82	yes
F	14	Ewing sarcoma	1.63	3.57	yes
F	6	Wilms tumor	0.81	3.91	yes
F	18	Ewing sarcoma	0.81	2.97	yes
M	9	Alveolar rhabdomyosarcoma	3.26	3.62	yes
F	5	Diffuse intrinsic pontine glioma	2.44	2.85	yes
M	10	Ewing sarcoma	1.63	0.17	yes
F	1	Neuroblastoma	1.63	7.53	yes
F	10	Ewing sarcoma	7.33	4.82	yes
M	20	Glioblastoma H3G34R+	7.33	8.02	yes
F	2	Neuroblastoma	5.70	6.33	yes
F	1	Embryonal rhabdomyosarcoma	1.63	6.39	yes
M	3	Burkitt lymphoma	10.59	6.08	yes
M	7	Burkitt lymphoma	19.55	14.18	yes
M	18	Glioblastoma	265.56	269.75	yes
F	10	Low-grade astroblastoma	1.63	1.83	no
M	4	Adrenocortical carcinoma	0.00	0.88	no
M	15	Hepatocellular carcinoma	2.44	3.59	no
M	3	Epithelial hepatoblastoma	2.44	0.46	no
M	5	Embryonal rhabdomyosarcoma	6.52	3.68	no
M	3	Embryonal rhabdomyosarcoma	4.07	5.71	no
F	7	Glioblastoma	0.81	4.48	no
M	1	Anaplastic ependymoma	1.63	6.65	no
F	4	Diffuse intrinsic pontine glioma H3K27M+	9.78	5.39	no

* M1—Method 1 for calculation of TMB excluding synonymous variants and indels.

**Table 3:** WES-based TMB values during tumor progression in nine patient case cohorts.

Gender	Age at Diagnosis	Diagnosis	Diagnosis/Relapse	Year of Biopsy	TMB (WES M1 *) Real-Life
F	9	Supratentorial ependymoma	local relapse	2016	2.31
			local relapse	2018	3.88
F	1	Neuroblastoma	metastatic relapse	2017	7.53
			metastatic relapse	2018	3.17
M	11	Ewing sarcoma	primary tumor	2017	2.57
			local relapse	2018	4.19
M	5	DIPG	primary tumor	2015	2.51
			local relapse	2018	6.68
F	10	LG astroblastoma	primary tumor	2017	1.83
			local relapse	2018	3.05
M	3	Epithelial hepatoblastoma	primary tumor	2016	0.46
			local relapse	2018	2.48
F	2	Ependymoma	primary tumor	2014	10.38
			metastatic relapse	2018	10.53
M	18	Osteosarcoma	metastatic relapse	2018	7.47
			metastatic relapse	2018	8.10
M	1	Infantile myofibromatosis	metastatic relapse	2015	2.08
			metastatic relapse	2018	1.88

* M1—Method 1 for calculation of TMB excluding synonymous variants and indels.

**Table 4:** WES-based TMB values during tumor progression in nine patient case cohorts.

	TMB—M1 * In Silico (Number of Cases Above Cut-Off)			TMB—M2 ** In Silico (Number of Cases Above Cut-Off)		
**Cut-off for ICIs Indication (mut/Mb)**	≥5	≥10	≥20	≥5	≥10	≥20
WES	30	8	4	75	25	7
MSK-IMPACT	23	6	4	61	12	6
F1CDx	24	7	5	42	11	6

* M1—Method 1 for calculation of TMB excluding synonymous variants and indels; ** M2—Method 2 for calculation of TMB including synonymous variants and indels; ICIs—immune checkpoint inhibitors.

**Table 5:** Number of patients stratified according to their diagnoses and baseline clinical data.

Diagnosis	Number of Patients	Gender Ratio (F/M)	Age Median	Age (Min–Max)	Type of Sample Ratio (Primary Tumor/Local or Metastatic Relapse)
High-grade glioma H3K27M+	12	8/2	9	4–20	12/0
Rhabdomyosarcoma	11	7/4	5	0–18	6/5
Ewing sarcoma	11	6/5	11	8–18	2/9
Neuroblastoma	10	6/4	2	1–8	1/9
Ependymoma	10	6/4	5.5	1–16	4/6
Non-rhabdomyosarcoma soft-tissue sarcomas	8	2/6	12	8–19	0/8
High-grade glioma H3K27M wt	6	0/6	16	8–23	5/1
Low-grade glioma	6	1/5	9.5	3–19	1/5
Osteosarcoma	5	4/1	18	14–28	0/5
Burkitt lymphoma	3	0/3	7	3–12	0/3
Medulloblastoma	3	0/3	4	2–5	1/2
Fibromatosis	3	1/2	17	13–20	1/2
Wilms tumor	2	1/1	8.5	6–11	1/1
Renal cell carcinoma	2	1/1	13.5	9–18	1/0
Adrenocortical carcinoma	1	F	4	-	primary tumor
Choroid plexus carcinoma	1	M	1	-	primary tumor
Hepatocellular carcinoma	1	M	15	-	primary tumor
Lung adenocarcinoma	1	F	15	-	metastatic relapse
Familiar infantile myofibromatosis	1	M	1	-	primary tumor
Myeloid sarcoma	1	F	5	-	primary tumor
Undifferentiated embryonal tumor of spinal canal	1	M	2	-	primary tumor
CNS germ cell tumor	1	M	11	-	local relapse
Epithelial hepatoblastoma	1	M	3	-	primary tumor
Spindle cell hemangioendothelioma	1	M	6	-	primary vascular malformation
Fibrodysplasia ossificans progressiva	1	F	1	-	primary tumor
Hepatosplenic T-lymphoma	1	M	17	-	diagnostic aspiration/bone marrow
Multiple system Langerhans cell histiocytosis	1	M	2	-	metastasis
Gastrointestinal stromal tumor	1	F	14	-	metastatic relapse
